# Does the effect of early manual lymphatic drainage during postoperative hospital stay prevent the development of lower limb lymphedema after gynecologic cancer surgery? A real-world cohort study

**DOI:** 10.7717/peerj.21275

**Published:** 2026-05-18

**Authors:** Lili Wu, Xinran Chen, Donglian Mo, Chunli Duan, Xiping Wu, Xin Chang

**Affiliations:** 1 Department of Gynecologic Oncology, Guangxi Medical University Cancer Hospital, Nanning, Guangxi, China; 2 Department of Nursing, The First People’s Hospital of Qinzhou, Qinzhou, Guangxi, China; 3 Department of Nursing, Guangxi Hydropwer Hospital, Nanning, Guangxi, China

**Keywords:** Gynecological cancers, Lymphedema, Manual lymphatic drainage, Propensity score matching, Retrospective cohort study

## Abstract

**Objective:**

To investigate how early manual lymphatic drainage can prevent lower limb lymphedema (LLL) following real-world gynecological cancer surgery.

**Methods:**

A total of 342 patients with gynecological cancers underwent radical surgery at a Guangxi cancer hospital between January 3 and February 15, 2025 were included. In order to ascertain the implementation of preventive manual lymphatic drainage (MLD) following surgery, patients were categorized into a prophylaxis group and a control group according to their selection. To control for potential confounding factors, a 1:1 propensity score-matching (PSM) method was used. Using the gynecological lymphedema questionnaire (GLQ) at least 6 months after surgery, we investigated the incidence of LLL and the status of preventive strategies after the intervention.

**Results:**

After PSM, 111 pairs of score-matched patients were generated. The prophylaxis group’s LLL incidence was 10.81%, substantially lower than the control group’s 21.62% (*P* = 0.04). 15.6% of patients in the control group and 38.9% of patients in the prophylaxis group engaged in preventive activities (*P $\lt$* 0.001). 71.7% of patients with GCLQ scores ≥ 4 took action to prevent LLL progression. The prophylaxis group showed a significantly reduced risk of LLL (risk ratio, 0.50; 95% confidence interval (CI) [0.263–0.949]; *P* = 0.034).

**Conclusion:**

The occurrence of postoperative lower limb lymphedema is reduced in gynecologic cancer patients who undergo early manual lymphatic drainage. This offers compelling evidence to inform clinical treatment decisions.

## Introduction

Gynecological cancers (GCs) include cervical cancer, endometrial cancer, ovarian cancer, and fallopian tube cancer, which are common cancers in women and pose a serious threat to their life and health (Dai et al., 2021). The most recent global cancer statistics report indicates that in 2022, approximately 1.47 million new cases of gastric cancers were recorded, alongside 680,000 fatalities attributed to the disease. The incidence of GCs reached 30.3 per 100,000, and the mortality rate hit 13.2 per 100,000 (Zhu et al., 2024).

The primary therapeutic approaches for GCs encompass surgical intervention and extensive treatment modalities, including radiation therapy, which significantly prolong patient survival yet simultaneously elevate the likelihood of post-treatment complications, such as lymphedema (Leandersson et al., 2021). One of the most frequent side effects after surgery and thorough treatment for GCs is secondary lower limb lymphedema (LLL). While the general incidence of LLL associated with GC treatment hovers around 25%, it may surpass 50% in specific cohorts, particularly among individuals undergoing radiation and pelvic lymph node dissection (Wang et al., 2020; Yost et al., 2014).

Leg swelling (affecting the ankles, calves, thighs, and perineum) can cause severe discomfort for patients with LLL. This discomfort can manifest as tight, heavy, or numb skin (Salani et al., 2014). Although early-stage lymphedema can be reversed, if treatment is delayed, it worsens and can result in stage 3 lymphedema, coexisting infections, or even amputation of the afflicted limb, which can significantly lower patients’ quality of life and psychological health (Zhang et al., 2022). There are studies from Sweden, demonstrating that early compression alone after breast cancer reduces the rate of Lymphedema significantly (Johansson et al., 2023). Compression stockings enhance venous and lymphatic return in the lower extremities *via* moderate pressure, aiding in the prevention and alleviation of mild edema resulting from prolonged standing, sitting, or venous insufficiency; nevertheless, they cannot entirely avert all forms of swelling. Thus, early postoperative prevention and care are essential for reducing the incidence and course of LLL (Liao et al., 2023).

Although manual drainage as part of complete decongestive therapy (CDT) has been shown to reduce the incidence of lymphedema, further research is needed to confirm its efficacy when performed alone (Tumkaya & Seven, 2025). The early use of manual lymphatic drainage or thorough decongestive therapy has also been shown to be effective in avoiding LLL in related prospective randomized controlled trials (Wu et al., 2021). However, the usefulness of early manual drainage in real-world applications remains unknown, as postoperative health education and self-management are directly linked to the onset and progression of lymphedema.

Real-world studies can more precisely depict the efficacy of nursing interventions than carefully planned randomized trials, providing fresh perspectives and methods for their use and improvement (Patorno, Schneeweiss & Wang, 2020). While manual lymphatic drainage is a component of complex decongestive therapy, its standalone efficacy in real-world settings remains underexplored. For this reason, we carried out a retrospective cohort study in the real world. We examined the effect of early manual drainage on preventing LLL after surgery for GCs, using 1:1 propensity score matching to account for differences in baseline clinical parameters between groups.

## Methods and materials

This research was approved by the Ethics Committee of Guangxi Zhuang Autonomous Region Cancer Prevention and Treatment Research Institute (Ethical approval number: KY2024428). We were in accordance with the 1975 Helsinki declaration and its later amendments. Written informed consent was obtained from the patient.

### Data source

Clinical data concerning gastric cancer patients hospitalized to the Department of Gynecology and Oncology at Guangxi Medical University Affiliated Cancer Hospital, who underwent radical surgery between June 2021 and June 2024, was retrospectively collected *via* the electronic medical record system. These were consecutive patients, and all eligible patients during the specified study period were included in this analysis. No eligible patients were excluded or missed during enrollment.

### Inclusion and exclusion criteria

Inclusion: (1) Endometrial, cervical, ovarian, or fallopian tube cancer with pathological confirmation; (2) Radical surgery (open or laparoscopic).

Exclusion: (1) Deep vein thrombosis following surgery; (2) Concurrent primary malignancies or recurrence.

### Grouping and interventions

On postoperative day 2, the attending physician evaluated the patient for ambulation. Patients reporting symptoms such as lower limb weakness or stiffness received counseling on the causes, goals, procedure, and cost of early preventive manual lymphatic drainage (MLD). With the patient’s consent, MLD was prescribed and relevant examinations were ordered. The actual MLD intervention commenced on postoperative day 3. Patients were divided into two groups based on their decision to receive early preventive MLD—a decision made following individual consultation on the procedure’s efficacy and cost. Those who opted for MLD formed the prophylaxis group, while those who did not formed the concurrent control group.

**Control group**: Following surgery, patients received regular instruction and advice from the nurse regarding pertinent preventive measures, such as skin care, including wearing loose cotton underwear, avoiding mosquito bites, and using moisturizer after every shower. Avoid lifting heavy objects, sitting or standing for extended periods, and engaging in strenuous functional exercises. Seek prompt diagnosis and treatment at specialized clinics linked to lymphedema if lower limb symptoms like pain, swelling, and numbness appear.

**Prophylaxis group**: In addition to control group measures, the lymphedema therapist performed early manual lymphatic drainage on the patient on the third day following surgery, after ultrasonography confirmed no deep vein thrombosis in either lower limb. After being put in a supine position, the patient was allowed to relax completely. To activate the lymphatic system, the lymphedema therapist used a static rotation technique to stimulate the lymph nodes in the neck, axilla, abdomen, and groin. To encourage lymphatic fluid reflux from the distal end of the limb to the proximal end, four fundamental lymphatic drainage procedures were then employed: rotation, pumping, shoveling, and static circular movements. Simple manual drainage techniques were taught during the procedure, and the patient was informed of the importance of performing lymphatic drainage at home. The manual lymphatic drainage treatment consisted of five sessions, 40 min each, in the hospital, with self-treatment afterwards. The patient did not present with lymphedema during the early postoperative assessment on day 2; consequently, the patient declined the use of compression stockings as they were not deemed necessary.

### Follow-up and observation indicators

Patient data were systematically extracted from the institutional electronic medical record (EMR) system. Subsequently, utilizing a catalog of patients who had received preventive manual lymphatic drainage (as documented by the certified lymphedema therapist), the overall cohort was delineated into two distinct groups: a prevention intervention group and a standard care control group. For every group, the researchers will follow up by phone. The median duration from surgery to this evaluation was 9 months in both the intervention and control groups.

#### General information survey form

The form was meticulously crafted by the researchers, encompassing clinical data such as disease type, stage, the quantity of lymph nodes excised, and adjuvant treatment modalities. Additionally, it incorporated general information including age, preoperative BMI, the occurrence and frequency of preventive manual drainage, and the existence or lack of home preventive practices, which included methods like simple manual drainage, plantar pumps, and elastic stockings.

#### Gynecological cancer lymphadema questionnaire

The Gynecological cancer lymphadema questionnaire (GCLQ) is a self-assessment tool designed for patients, developed by American researchers (Carter et al., 2010). It aims to evaluate symptoms including lower limb edema, heaviness, localized swelling, discomfort, infection, numbness, and functional activity that individuals have encountered over the past 4 weeks (Carter et al., 2010). The GCLQ questionnaire comprises 20 items, yielding a total score ranging from 0 to 20 points. Among these, seven key symptom items are scored dichotomously (yes = 1, no = 0). A score greater than four points on these seven items is indicative of a diagnosis of lower limb lymphedema. Using this threshold, the instrument demonstrates a sensitivity of 0.929 and a specificity of 0.833. This research involved the administration of the GCLQ *via* telephone, focusing on symptom-defined lymphedema instead of clinically confirmed cases of lymphedema.

### Statistical analysis

SPSS 26.0 (IBM Corp., Armonk, NY, USA) was used to analyze the data. Frequency serves as a representation of count data, while the chi-square test or Fisher’s exact probability test facilitates group comparisons. Measurement data adhering to a normal distribution is expressed as mean ± SD, whereas data that deviates from this norm is represented by median (P25, P75). For intergroup comparisons, independent sample t-tests or non-parametric tests are employed for analysis. To reduce confounding and minimize selection bias, we performed propensity score matching (PSM) using a 1:1 nearest-neighbor algorithm with a caliper width of 0.02. Propensity scores were estimated using a logistic regression model that included baseline characteristics as covariates. These covariates comprise age, body mass index (BMI), pathological stage, initial tumor type, postoperative duration, radiation therapy, chemotherapy, and the number of lymph node resections. This matching process created balanced cohorts by mitigating imbalances in observed confounders between the prophylaxis and control groups. We evaluated the success of the matching procedure by calculating standardized mean differences (SMDs) for all covariates. A difference is considered statistically significant if *P* < 0.05.

## Results

### Case inclusion status

Out of the 147 patients in the prophylaxis group who met the inclusion criteria, twelve were lost to follow-up, one succumbed, two experienced thrombosis, and one faced a recurrence of a tumor. A total of 131 patients were subsequently incorporated into the prophylaxis group following their initial exclusion. A total of 249 patients in the control group satisfied the inclusion criteria, which encompassed three fatalities, two instances of thrombosis, five occurrences of tumor recurrence, and 28 individuals lost to follow-up. A total of 211 patients were subsequently incorporated into the control group after the exclusion process was completed.

Before PSM, the two groups were comparable with respect to age, BMI, postoperative time, tumor type, and tumor stage (all *P* > 0.05). Nonetheless, significant differences were identified in the administration of chemotherapy, the delivery of radiotherapy, and the quantity of lymph nodes excised (*P* < 0.05). After performing 1:1 PSM, 111 patients in the prophylaxis group were successfully matched with 111 patients in the control group. The post-matching analysis revealed that the baseline characteristics were effectively balanced across the two groups, with no statistically significant differences observed (all *P* > 0.05). The detailed baseline characteristics before and after matching are presented in Table 1.

**Table 1:** Comparison of clinical characteristics between two groups before and after propensity score matching (Continuous variables: mean ± SD or median (P25, P75); Categorical variables: *n* (%)).

Item	Before matching		*P*	After matching		*P*
	Prophylaxis group (131)	Control group (211)		Prophylaxis group (111)	Control group	
Age (years)	50.15 ± 8.93	51.42 ± 9.67	0.89	49.87 ± 8.09	49.94 ± 8.86	0.96
Body mass index (kg/m²)	22.42 ± 2.96	23.03 ± 3.24	0.84	22.88 ± 2.59	22.98 ± 3.12	0.80
Postoperative time (months)	9 (6, 13)	9 (6, 13)	0.335	9 (6, 13)	10 (7, 15)	0.15
Primary tumor type						
Cervical cancer	75 (57.25)	131 (62.08)		67 (60.36)	79 (71.17)	
Endometrial cancer	41 (31.29)	43 (20.39)	0.07	33 (29.73)	21 (18.92)	0.13
Ovarian cancer	13 (9.92)	27 (12.79)		9 (8.11)	6 (5.41)	
Fallopian tube cancer	2 (1.53)	10 (4.74)		2 (1.80)	5 (4.50)	
Pathological type						
I	66 (50.38)	101 (47.88)	0.24	57 (51.35)	58 (52.25)	0.71
II	34 (25.95)	58 (27.48)		27 (24.32)	32 (28.82)	
III	27 (20.61)	35 (16.59)		23 (20.72)	17 (15.32)	
IV	4 (3.05)	17 (8.06)		4 (3.60)	4 (3.60)	
Chemotherapy						
Yes	102 (77.86)	119 (56.40)	<0.001	84 (75.68)	84 (75.68)	1
No	29 (22.14)	92 (43.60)		27 (24.32)	27 (24.32)	
Radiotherapy						
Yes	57 (43.51)	53 (25.12)	<0.001	41 (36.94)	43 (38.74)	0.89
No	74 (56.49)	158 (74.88)		70 (63.06)	68 (61.26)	
Number of lymph nodes removed	30.75 ± 13.69	19 (0, 31)	<0.001	29.18 ± 11.41	28.39 ± 13.44	0.64

### LLL incidence

Before PSM, the incidence of LLL was 12.21% in the prophylaxis group and 20.85% in the control group, indicating a statistically significant difference between the two groups (*P* = 0.04). After matching, the occurrence of LLL was observed to be 10.81% in the prophylaxis group compared to 21.62% in the control group. Table 2 indicates that there was a statistically significant difference between the two groups (*P* = 0.03). The prophylaxis group showed a significantly reduced risk of LLL (risk ratio, 0.50; 95% CI [0.263–0.949]; *P* = 0.034).

**Table 2:** Incidence of lymphedema in two groups of patients before and after propensity score matching, *n* (%).

Item	Before matching		χ2	*P*	After matching		χ2	*P*
	Prophylaxis group (131)	Control group (211)			Prophylaxis group (111)	Control group (111)		
GCLQ ≥ 4	16 (12.21)	44 (20.85)	4.17	0.04	12 (10.81)	24 (21.62)	4.77	0.03
GCLQ $\lt$ 4	115 (87.78)	167 (79.15)			99 (89.19)	87 (78.38)		

### Current status of early prevention and treatment of LLL

**Figure 1:**
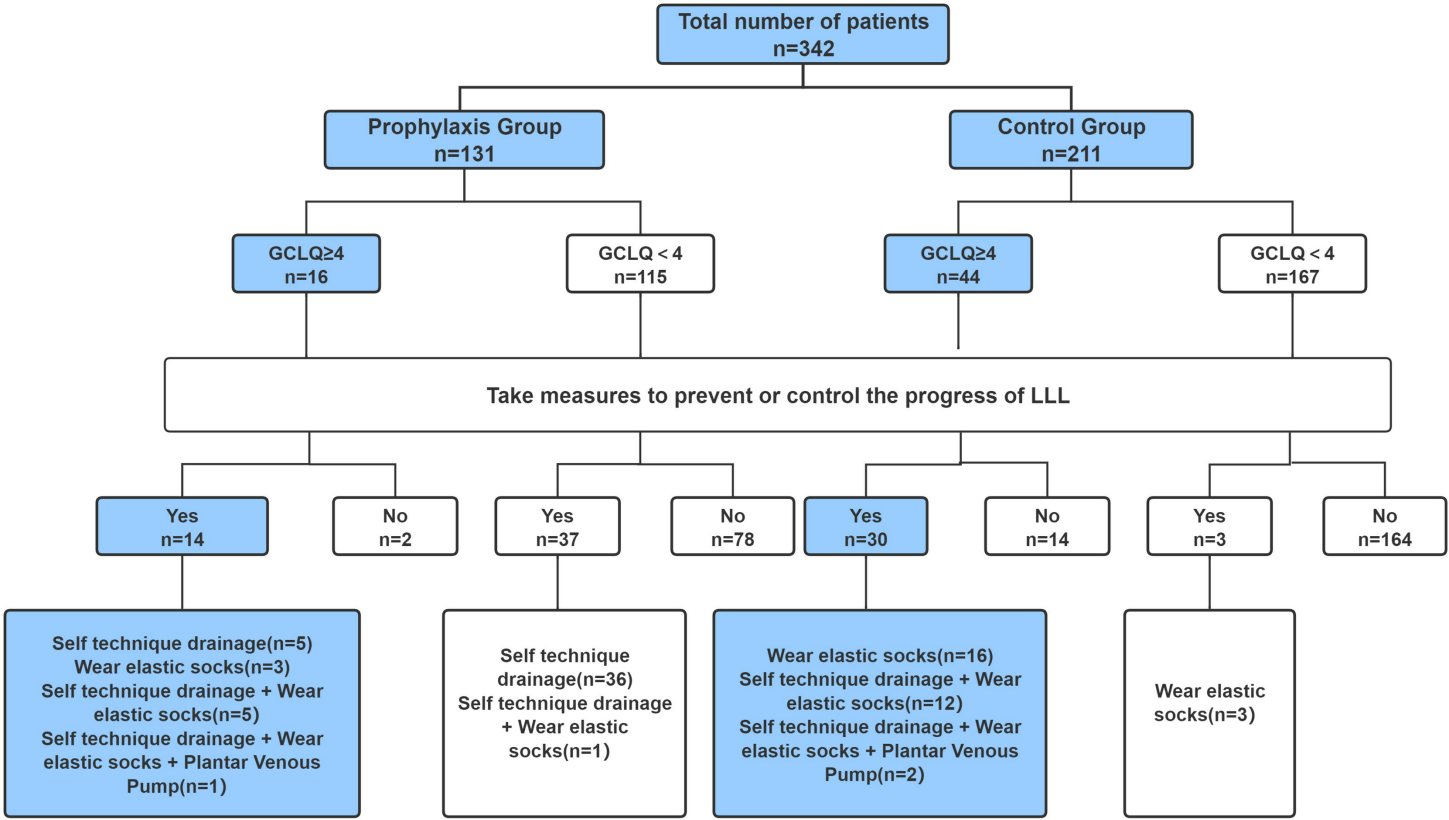
Types of prevention and treatment strategies adopted by two groups of patients.

Regarding home-based prevention and treatment behaviors, 51 patients (38.9%) in the prophylaxis group and 33 patients (15.6%) in the control group reported participating in these measures. A total of 60 patients had a GCLQ score of ≥4, indicating symptom-defined lymphedema. Among these, 43 patients (71.7%) had adopted measures to prevent or manage the progression of lower limb lymphedema (LLL). In this symptomatic subgroup (GCLQ ≥ 4), the percentage of patients participating in preventive behaviors was significantly greater in the prophylaxis group (87.5%) than in the control group (68.2%). The specific types of preventive and therapeutic strategies adopted by patients in both groups are detailed in Fig. 1.

## Discussion

The prevention and management of postoperative lower-limb lymphedema in GCs have consistently been a focus for medical professionals. Boccardo et al. (2016) showed that performing lymphatic vein anastomosis using microsurgical techniques at the conclusion of inguinal lymph node dissection can effectively prevent lower limb lymphedema (Boccardo et al., 2016). However, it is indisputable that both the surgical duration and expenses would significantly escalate, and its efficacy as a standard procedure for lymphedema prevention remains contentious. Wu et al. (2021) illustrated that the timely implementation of a holistic approach, which includes manual lymphatic drainage, pressure therapy (primarily involving compression stockings and foot pump therapy), exercise training, and skin care, can significantly reduce the incidence of edema, prolong the duration of edema-free survival, and mitigate symptoms such as limb heaviness and pain. The emergence and progression of LLL is a protracted process, and while early postoperative intervention may enhance short-term clinical outcomes, its practical implementation requires further investigation (Wong et al., 2022).

PSM’s core function is to create comparison groups that are statistically similar on all observed pre-treatment characteristics (confounders). By matching individuals with similar propensity scores, the probability of receiving treatment given their observed covariates, it balances these confounders across groups. This reduces the bias that occurs when treated and control units differ systematically at baseline. This study employed the PSM approach to balance baseline data between groups for comparability, demonstrating that the incidence of LLL in the preventive group was significantly lower than in the control group, with the difference being statistically significant. This suggests that early manual drainage might successfully diminish the occurrence of lower limb lymphedema following GCs surgery, even in practical settings. In this study, the incidence of LLL among patients in the control group receiving standard nursing interventions was comparable to findings from several investigations (Wang et al., 2020; Bakar & Tugral, 2017), demonstrating that early manual draining for postoperative patients with GCs is more efficacious in preventing lower limb lymphedema than traditional educational nursing in clinical practice. However, unmeasured confounders (*e.g*., patient compliance) might affect the results. Future research will employ more rigorous randomized controlled trials to further validate the findings of this study.

The majority of patients in the prophylaxis group reported substantial improvement in lower limb weakness and discomfort symptoms following 1–2 manual drainage sessions. However, the immediate effects of early manual drainage were not evaluated due to the lack of relevant quantitative assessment tools. The potential explanation for the aforementioned observations is that the reflux of lymphatic fluid is primarily facilitated by the pressure generated *via* muscle activity. Radical surgery partially obstructs the lymphatic reflux channel, and prolonged bed rest during and after the procedure, combined with inadequate muscle activity, causes the accumulation of lymphatic fluid in the surrounding tissues of the lower limbs, resulting in symptoms such as weakness and soreness in patients (Rostom & Salama, 2022). Early postoperative manual drainage involves exerting gentle pressure along the lymphatic veins using external effort to stimulate the lymph nodes and enhance their reabsorption ability. This facilitates the ingress of surplus fluid from adjacent tissues into the lymphatic system, thereby eliminating stagnant fluids, alleviating symptoms of edema and weakness, and fostering early patient mobilization (Liu et al., 2021). Furthermore, in standard nursing practice, despite the provision of pertinent educational information, postoperative patients typically experience pain and weakness, resulting in diminished capacity for information reception and comprehension. Over time, the important safeguards put in place during hospitalization are overlooked (Hajdarevic et al., 2022). Consequently, although some persons display pronounced symptoms of lower limb numbness and edema, they have not received adequate attention. Patients in the prophylaxis group, following early postoperative lower limb weakness, discomfort, and notable effects of manual drainage, exhibit considerable confidence in the technique. Throughout the procedure, individuals are able to more readily grasp the techniques and intensity of manual drainage, thereby deepening their comprehension and promoting ongoing involvement in effective self-preventive management in their daily lives. It can promptly address LLL symptoms, including numbness and depressed edema, by consulting certified lymphedema therapists and adopting strategies to avert edema advancement.

The occurrence of LLL is closely associated with treatment factors, including lymph node dissection and radiotherapy, in addition to the patient’s postoperative lifestyle choices, such as maintaining a single position for extended periods and resuming work shortly after surgery (Wong et al., 2022). Consequently, postoperative health education, self-management, and early patient identification are crucial in the prevention and treatment of LLL (Liao et al., 2023). The findings of this study reveal that postoperative patients continue to exhibit inadequate focus on the prevention of lower limb lymphedema. The proportion of individuals in both groups who actively participate in preventive strategies remains notably low, falling below 50%. Furthermore, a specific subset of patients (28.3%) who have already manifested LLL has not received sufficient attention, and measures to prevent further progression have yet to be established. In future clinical practice, nursing staff should formulate scientifically valid and effective prevention measures, supervise the implementation of patient interventions, significantly reduce the incidence of LLL, and prevent the progression of LLL.

Nevertheless, several limitations of this study must be acknowledged. First, the non-randomized group allocation based on patient choice introduces potential selection bias. It is noteworthy that, given the self-funded nature of the MLD intervention, there may exist systematic differences between patients in the prophylaxis group and those in the control group regarding socioeconomic status, health awareness, and self-care behaviors. The aforementioned factors may exert independent influences on postoperative recovery outcomes, thereby constituting a potential source of confounding beyond our complete control, notwithstanding the application of PSM. Future studies should consider randomized controlled designs or collect detailed data on socioeconomic covariates to better address this limitation. Second, the absence of objective limb measurements means our findings pertain to patient-reported lymphedema symptoms, which may not fully correspond to clinically diagnosed lymphedema. Third, a significant drawback is the absence of a comparison between patients who lost to follow-up and those retained, as well as an evaluation of potential differences in follow-up rates between the groups. The absence of this comparison introduces uncertainty regarding potential attrition bias and its impact on generalizability. Future studies should systematically track loss to follow-up to mitigate this issue. Finally, the follow-up period was relatively short, which may not have captured the long-term incidence or progression of lymphedema. Extended follow-up is needed to assess the durability of the intervention’s effects. In addition, despite adjusting for measured confounders, we cannot rule out unmeasured confounding. Postoperative exercise habits, occupational physical demands, compliance with home-based preventive strategies, and various behavioral or lifestyle factors were not thoroughly documented and may have impacted the outcomes. In conclusion, this study utilized PSM to balance baseline data between groups and achieve comparability, examining the effectiveness of early manual drainage in preventing lower limb lymphedema after gynecological malignancy surgery in a practical setting. The findings indicated that early manual drainage can reduce the risk of lower limb lymphedema in individuals with GCs after surgery. This study constitutes a retrospective analysis of real-world data. Due to the limitations inherent in this research and the lack of adequate follow-up, it is impractical to account for all confounding variables, such as the characteristics and extent of the job, assessment of physical activity, among others. Furthermore, individuals in the prophylaxis cohort continue to exhibit a notable incidence of lymphedema. As a result, additional influencing factors will be integrated into comprehensive prospective randomized controlled studies in the future to further explore effective strategies for alleviating secondary lower limb lymphedema.

## Supplemental Information

10.7717/peerj.21275/supp-1 Supplemental Information 1 Raw data.

## Additional Information and Declarations

### Data Availability

The following information was supplied regarding data availability:

The raw data is available in the Supplemental File.

## References

[ref-1] Bakar Y, Tugral A (2017). Lower extremity lymphedema management after gynecologic cancer surgery: a review of current management strategies. Annals of Vascular Surgery.

[ref-2] Boccardo F, Valenzano M, Costantini S, Casabona F, Morotti M, Sala P, De Cian F, Molinari L, Spinaci S, Dessalvi S, Campisi CC, Villa G, Campisi C (2016). LYMPHA technique to prevent secondary lower limb lymphedema. Annals of Surgical Oncology.

[ref-3] Carter J, Raviv L, Appollo K, Baser RE, Iasonos A, Barakat RR (2010). A pilot study using the Gynecologic Cancer Lymphedema Questionnaire (GCLQ) as a clinical care tool to identify lower extremity lymphedema in gynecologic cancer survivors. Gynecologic Oncology.

[ref-4] Dai Y, Wang J, Zhao L, Wang Z (2021). BRCA1/2mutations and survival of high-grade endometrioid endometrial cancer. Gynecology and Obstetrics Clinical Medicine.

[ref-5] Hajdarevic S, Fallbjork U, Fransson P, Astrom S (2022). Need of support perceived by patients primarily curatively treated for breast, colorectal, or prostate cancer and close to discharge from hospital: a qualitative study. Journal of Clinical Nursing.

[ref-6] Johansson K, Blom K, Nilsson-Wikmar L, Brogårdh C (2023). Early intervention with a compression sleeve in mild breast cancer-related arm lymphedema: a 12-month prospective observational study. Cancers.

[ref-7] Leandersson P, Hogberg T, Dickman PW, Malander S, Borgfeldt C (2021). Incidence and survival of epithelial ovarian, fallopian tube, peritoneal, and undesignated abdominal/pelvic cancers in Sweden 1960–2014: a population-based cohort study. BMC Cancer.

[ref-8] Liao X, Cao G, Yang L, Wang C, Tian C (2023). Postoperative effectiveness of comprehensive nursing intervention for lymphedema in gynecological cancer: a controlled study. Alternative Therapies in Health and Medicine.

[ref-9] Liu F, Liu NF, Wang L, Chen J, Han L, Yu Z, Sun D (2021). Treatment of secondary lower limb lymphedema after gynecologic cancer with complex decongestive therapy. Lymphology.

[ref-10] Patorno E, Schneeweiss S, Wang SV (2020). Transparency in real-world evidence (RWE) studies to build confidence for decision-making: reporting RWE research in diabetes. Diabetes, Obesity and Metabolism.

[ref-11] Rostom EH, Salama AB (2022). Vodder manual lymphatic drainage technique versus Casley-Smith manual lymphatic drainage technique for cellulite after thigh liposuction. Advances in Dermatology and Allergology.

[ref-12] Salani R, Preston MM, Hade EM, Johns J, Fowler JM, Paskett EP, Katz ML (2014). Swelling among women who need education about leg lymphedema: a descriptive study of lymphedema in women undergoing surgery for endometrial cancer. International Journal of Gynecological Cancer.

[ref-13] Tumkaya MN, Seven M (2025). Interventions for prevention and management of gynecological cancer-related lower limb lymphedema: a systematic scoping review. Seminars in Oncology Nursing.

[ref-14] Wang X, Ding Y, Cai HY, You J, Fan FQ, Cai ZF, An P (2020). Effectiveness of modified complex decongestive physiotherapy for preventing lower extremity lymphedema after radical surgery for cervical cancer: a randomized controlled trial. International Journal of Gynecological Cancer.

[ref-15] Wong M, Eaton PK, Zanichelli C, Moore C, Hegarty C, MacDonald N (2022). The prevalence of undiagnosed postoperative lower limb lymphedema among gynecological oncology patients. European Journal of Surgical Oncology.

[ref-16] Wu X, Liu Y, Zhu D, Wang F, Ji J, Yan H (2021). Early prevention of complex decongestive therapy and rehabilitation exercise for prevention of lower extremity lymphedema after operation of gynecologic cancer. Asian Journal of Surgery.

[ref-17] Yost KJ, Cheville AL, Al-Hilli MM, Mariani A, Barrette BA, McGree ME, Weaver AL, Dowdy SC (2014). Lymphedema after surgery for endometrial cancer: prevalence, risk factors, and quality of life. Obstetrics & Gynecology.

[ref-18] Zhang H, Kong W, Han C, Liu T, Li J, Song D (2022). Current status and progress in the treatment of lower limb lymphedema after treatment of gynecological oncology. Lymphatic Research and Biology.

[ref-19] Zhu B, Gu H, Mao Z, Beeraka NM, Zhao X, Anand MP, Zheng Y, Zhao R, Li S, Manogaran P, Fan R, Nikolenko VN, Wen H, Basappa B, Liu J (2024). Global burden of gynaecological cancers in 2022 and projections to 2050. Journal of Global Health.

